# Research progress on risk factors and clinical management of *Ureaplasma* Species infection in preterm infants

**DOI:** 10.3389/fped.2026.1845719

**Published:** 2026-05-18

**Authors:** Shanshan Li, Jili Zhang, Caiyun Miao, Linyan Zhu, Jiena Shi, Qingmei Sun, Longhui Shen

**Affiliations:** 1 Department of Pharmacy, The Affiliated Women and Children’s Hospital of Ningbo University, Ningbo, Zhejiang, China; 2 Health Science Center, Ningbo University, Ningbo, Zhejiang, China

**Keywords:** clinical management, preterm infants, research progress, risk factors, *Ureaplasma* species

## Abstract

*Ureaplasma species* is a common opportunistic pathogen transmitted from mother to fetus. *Ureaplasma* spp. infection is closely associated with severe complications in preterm infants, such as pneumonia, bronchopulmonary dysplasia (BPD), and sepsis, significantly impacting prognosis. Early identification of potential risk factors for *Ureaplasma* spp. infection in preterm infants and timely implementation of effective management strategies are of critical importance. This article mainly reviews the research progress on risk factors and clinical management of *Ureaplasma* spp. infection in preterm infants, aiming to provide a reference for optimizing clinical identification strategies for *Ureaplasma* spp. infection in preterm infants and reducing the incidence of adverse outcomes.

## 1 Introduction

*Ureaplasma* spp. belong to the family Mycoplasmataceae within the order Mycoplasmoidales. They are thought to be the smallest free-living, self-replicating organisms. As they do not have a cell wall, they are limited to a parasitic existence in eukaryotic cells (1). In 2002, *Ureaplasma* spp. was subdivided into 2 separate species or bio variations (biovars): *Ureaplasma urealyticum* (Uu) and *Ureaplasma parvum* (Up) (2). Epidemiological evidence indicates that the positivity rate of *Ureaplasma* spp. in pregnant women is approximately 82%. The average colonization rate in maternal genitalia ranges from 40% to 80%, showing an increasing trend as gestational age (GA) decreases (3). The results showed that the *Ureaplasma* spp. positivity rate among preterm infants with a GA < 26 weeks within 1 month of birth was as high as 65%, whereas among those with a GA > 26 weeks, the positivity rate was only 31% (4). *Ureaplasma* spp. readily colonizes the respiratory tract, rectum, and genitourinary mucosal surfaces. Preterm infants are particularly susceptible to *Ureaplasma* spp. colonization and infection due to their underdeveloped immune systems and immature skin and mucosal barriers (5). Mechanistically, *Ureaplasma* spp. evades host immune defenses by degrading IgA antibodies and antagonizing nutritional immunity. Furthermore, it regulates cellular gene expression and promotes inflammatory cytokines, such as IL-6 and IL-1β. These processes exacerbate inflammatory responses, leading to multi-organ damage (6–9). *Ureaplasma* spp.-driven immunological implications appear to depend on pathogen virulence and duration of pathogen exposure as well as on host immune function, genetic background, and maturity (10). Clinically, *Ureaplasma* spp. infection is a significant cause of congenital pneumonia in preterm infants. It is also strongly associated with respiratory distress syndrome and BPD (7). Additionally, *Ureaplasma* spp. can invade the bloodstream and cross the blood-brain barrier, resulting in sepsis, meningitis, intraventricular hemorrhage, and hydrocephalus (1, 7). Infection also elevates the risk of necrotizing enterocolitis and retinopathy. These severe complications contribute to increased morbidity and mortality, imposing a substantial burden on families and society (7). Therefore, *Ureaplasma* spp. infection poses a serious threat to preterm infant health, necessitating enhanced early clinical monitoring and improved intervention strategies.

However, the clinical manifestations following *Ureaplasma* spp. infection in preterm infants are diverse and non-specific. Coupled with the special requirements of *Ureaplasma* spp. for specimen transport conditions and culture media, detection techniques are technically challenging and have limited sensitivity. These factors can lead to delayed clinical recognition or misdiagnosis, thereby affecting the timing of early intervention and prognosis for preterm infants (1, 11). Existing studies have shown that *Ureaplasma* spp. infection in preterm infants is associated with risk factors such as premature rupture of membranes(PROM) and GA (12). In recent years, early identification of risk factors for *Ureaplasma* spp. infection in preterm infants has become a research hotspot in neonatology. Additionally, constructing scientifically valid risk prediction models to assist clinical decision-making and management has drawn increasing attention (12). This article focuses on elaborating on the risk prediction and prevention strategies for *Ureaplasma* spp. infection in preterm infants, aiming to provide a theoretical basis for early clinical identification, effective intervention, and comprehensive prevention.

## 2 Risk factors for *Ureaplasma* spp. Infection in Preterm Infants

*Ureaplasma* spp. infection in preterm infants is not caused by a single factor but is a complex pathological process resulting from the combined influence of maternal high-risk factors and intrinsic infant factors (12). Therefore, identifying and elucidating the key risk factors is critical. Such efforts enable early warning and precise screening of preterm infants at high risk for *Ureaplasma* spp. infection, which in turn facilitates the development of targeted prevention and treatment strategies. This represents the essential first step in optimizing clinical interventions for preventing *Ureaplasma* spp. infection in preterm infants.

### 2.1 Maternal-Related risk factors

#### 2.1.1 Vaginal *Ureaplasma* spp. Colonization

Maternal colonization with *Ureaplasma* spp. is a critical determinant of preterm infants infection, primarily driven by vertical transmission. Evidence indicates that maternal vaginal colonization is a strong predictor of respiratory tract colonization in preterm infants (< 32 weeks), with an adjusted odds ratio of 7.8 (95% CI: 3.1–20.0) (12). Chun et al. further highlighted the clinical burden of high bacterial load, reporting a significantly higher incidence of BPD in infants born to mothers with high vaginal *Ureaplasma* spp. colonization (≥ 10⁴ CCU/mL) compared to those with low or no colonization (66% vs. 48% vs. 49%; *P* = 0.044). Notably, vertical transmission rates were elevated in the high colonization group (36.2%) (13). Pathophysiologically, high-density *Ureaplasma* spp. colonization may trigger intense inflammatory cascades and prothrombin messenger RNA expression in decidua cells, predisposing to PROM and subsequently facilitating vertical transmission (14).

#### 2.1.2 Placental *Ureaplasma* spp. Colonization

The placenta is a critical interface for maternal-fetal material exchange and immune regulation, and also serves as an important route for *Ureaplasma* spp. transmission from the maternal genital tract to the fetus (15). In a retrospective cohort study (*n* = 227), Yin et al. reported a polymerase chain reaction (PCR) detection rate of *Ureaplasma* spp. in placental specimens of 22.5%, and found that placental *Ureaplasma* spp. positivity was significantly positively correlated with the progression of fetal inflammatory response staging (*P* < 0.001). Among preterm infants with *Ureaplasma* spp. detected in both the placenta and neonatal urine, the incidence of severe neonatal complications reached 28.1% (16). Combined detection of *Ureaplasma* spp. in neonatal urine and the placenta helps identify preterm infants at the highest risk of adverse outcomes and may inform early risk stratification.

#### 2.1.3 Mode of delivery

The mode of delivery is a critical maternal factor influencing the risk of *Ureaplasma* spp. infection in preterm infants. Multiple studies confirm that while vaginal delivery significantly increases this risk, cesarean section offers a protective effect. For instance, Li et al. demonstrated that among infants born at 28–37 weeks, vaginal delivery was associated with a significantly higher risk of *Ureaplasma* spp. infection (OR = 3.11, 95% CI: 1.94–4.99) (17). In contrast, studies by Viscardi et al. (GA <33 weeks) and Sun et al. (GA <32 weeks) reported that cesarean section reduced infection risk, with ORs of 0.48 (95% CI: 0.30–0.78) and 0.18 (95% CI: 0.09–0.35), respectively (18, 19). Furthermore, these findings were reinforced by a multivariate analysis from Ji et al. which identified vaginal delivery as an independent risk factor for *Ureaplasma* spp. infection in infants with a GA < 32 weeks (aOR=3.55, 95% CI: 1.88–6.68) (20).

#### 2.1.4 Fetal membrane Status

Fetal membranes are a natural barrier that protects the fetus, and infection by reproductive tract pathogens is the most common cause of PROM. PROM and its duration are important risk factors for *Ureaplasma* spp. infection in preterm infants. Studies have shown that a higher percentage of vaginal deliveries (67.2%) and a higher incidence of premature rupture of membranes among pregnant mothers (45.8%) in the *Ureaplasma* spp.-positive group compared to the *Ureaplasma* spp.-negative group (5). Research by Sun et al. and Ji et al. both confirmed that PROM is associated with an increased risk of *Ureaplasma* spp. infection in infants with a GA < 32 weeks (OR = 2.10, 95% CI: 1.03–4.26; aOR=2.31, 95% CI: 1.31–4.07) (19, 20). Regarding the duration of PROM, Viscardi et al. further found that in infants with a GA < 33 weeks, a PROM duration > 72 h increased the risk of lower respiratory tract infection (OR = 2.15, 95% CI: 1.22–3.76) (18).

#### 2.1.5 Chorioamnionitis

Acute chorioamnionitis is often thought to be due to ascending infection—diffuse ascending colonization of the endometrial-chorionic potential space, with extension into the fetal membranes, the amniotic fluid, and ultimately the fetus (21). Clinical data show that chorioamnionitis is associated with an approximately 2-fold increased odds of neonatal adverse outcomes at < 34 weeks (22). In a study of preterm infants with GA < 32 weeks, Ji et al. found that chorioamnionitis was an independent risk factor for *Ureaplasma* spp. infection (aOR=4.53, 95% CI: 2.29–8.95) (20). This finding indicates that, as a significant pathological manifestation of intrauterine infection, chorioamnionitis significantly increases the likelihood of fetal exposure to *Ureaplasma* spp. and subsequent infection *in utero* or during delivery.

#### 2.1.6 Prenatal antibiotic exposure

Prenatal antibiotic exposure represents a key modifiable factor for improving clinical outcomes in preterm infants with *Ureaplasma* spp. infection. This is particularly relevant for maternal *Ureaplasma* spp.-positive cases, where prenatal intervention can reduce the incidence of complications in preterm infants. In a primate model, specific maternal antibiotic therapy can eradicate *Ureaplasma* spp. from the amniotic fluid and key fetal organs, thereby mitigating fetal lung injury (23). Kim et al. conducted a retrospective case-control study on preterm infants with GA ≤ 30 weeks from 2012 to 2016 (24). The results showed that there was no statistically significant difference in the overall incidence of BPD between the prenatal azithromycin treatment group and the non- *Ureaplasma* spp. colonization group (26.4% vs. 16.4%, *P* = 0.173). Another retrospective study of 260 preterm infants with birth weight < 1500 g further revealed the potential benefits of prenatal antibiotic therapy. The study found that in cases of maternal *Ureaplasma* spp. positivity, prenatal azithromycin treatment reduced the risk of moderate-to-severe BPD in preterm infants by approximately 65.7% (OR = 0.34, 95% CI: 0.14–0.86) (25).

The risk of *Ureaplasma* spp. infection in preterm infants is associated with the status of maternal vaginal and placental *Ureaplasma* spp. colonization. Screening for maternal *Ureaplasma* spp. bacterial load facilitates the early assessment of infection risk. Furthermore, this risk is influenced by multiple perinatal factors, including vaginal delivery, prolonged duration of PROM, chorioamnionitis, and lack of timely prenatal antibiotic exposure. Specifically, PROM causes the vaginal environment of the mother to become weakly alkaline, which creates a favorable environment for the survival of pathogens, that can infect the fetus through vertical transmission, and the risk of vertical transmission to the neonate is further increased by vaginal delivery (20). Additionally, *Ureaplasma* spp. can induce the release of related proteases, leading to collagen fragmentation and stimulating the production of inflammatory mediators such as IL-1β, IL-8, and TNF-α. These processes promote PROM and chorioamnionitis, subsequently infecting the fetus via the placenta or amniotic fluid and increasing the risk of ascending infection (5, 26).

### 2.2 Intrinsic risk factors in preterm infants

#### 2.2.1 GA

Previous studies have established that low GA is a critical risk factor for *Ureaplasma* spp. infection in preterm infants, with the risk increasing inversely with decreasing GA. Li et al. identified GA as an independent risk factor (OR = 0.85; 95% CI: 0.76–0.95) (17), and Sarwar et al. reported a similar negative correlation (OR = 0.74; 95% CI: 0.59–0.93) (27). Focusing on infants < 32 weeks GA, Viscardi et al. demonstrated that each additional week of GA was associated with a 32% reduction in the risk of lower respiratory tract *Ureaplasma* spp. infection (OR = 0.68, 95% CI: 0.59–0.78) (18). The inverse relationship between GA and *Ureaplasma* spp. risk is further supported by prevalence data: one study reported a *Ureaplasma* spp. colonization rate of 51.5% among infants < 28 weeks GA, which rose to 65% among those < 26 weeks (12).

#### 2.2.2 Birth weight

Accumulating evidence indicates that low birth weight is associated with an elevated risk of *Ureaplasma* spp. infection. Sarwar et al. reported a significant association between low birth weight and *Ureaplasma* spp. infection in preterm infants; the mean birth weight in the *Ureaplasma* spp.-positive group was significantly lower than that in the negative group (1,529 ± 501 g vs. 1,934 ± 601 g; *P* = 0.005) (27). Consistent with these findings, in a study of 561 *Ureaplasma* spp.-infected newborns, 301 (53.65%) had a birth weight < 2,500 g, including 18 (3.21%) with extremely low birth weight (ELBW, < 1,000 g) and 96 (17.11%) with very low birth weight (VLBW, 1,000–1,500 g). Furthermore, the mean birth weight of *Ureaplasma* spp.-positive neonates was significantly lower than that of the *Ureaplasma* spp.-negative group (2,426.4 ± 911.9 g vs. 2,701.5 ± 690.2 g; *P* < 0.001) (5).

#### 2.2.3 Inflammatory markers

A close relationship between postpartum inflammatory markers and *Ureaplasma* spp. infection in preterm infants has been reported. With respect to white blood cell (WBC) count, Viscardi et al. identified elevated WBC count upon admission as a significant risk factor for *Ureaplasma* spp. infection (OR = 1.05, 95% CI: 1.03–1.07) (18). Similarly, Sun et al. reported a comparable association between postpartum WBC count and *Ureaplasma* spp. infection (OR = 1.08, 95% CI: 1.03–1.13) (19). Regarding C-reactive protein (CRP), Li et al. demonstrated that elevated postpartum CRP levels served as an independent risk factor for *Ureaplasma* spp. infection in preterm infants (OR = 5.08, 95% CI: 1.91–13.49) (17). Moreover, Yang et al. observed significantly higher CRP levels in *Ureaplasma* spp.-positive preterm infants compared to their *Ureaplasma* spp.-negative counterparts (*P* < 0.05) (28).

#### 2.2.4 Genetic polymorphisms

Polymorphisms in host defense genes may serve as risk factors for *Ureaplasma* spp. infection in preterm infants. A study examined twenty-four tag single nucleotide polymorphisms (SNPs) from Toll-like receptor *TLR*1, *TLR*2, *TLR*4, and *TLR*6 genes among 298 preterm infants were assayed in 298 infants <33 weeks gestation who had serial respiratory cultures for *Ureaplasma* spp. and were evaluated for BPD (29). The findings indicated that the *TLR6* SNP rs5743827 was associated with a reduced risk of Ureaplasma respiratory colonization (OR = 0.54, 95% CI: 0.34–0.86) and a reduced risk of BPD (OR = 0.54, 95% CI: 0.31–0.95). Notably, the GG genotype at this locus exhibited a significant additive interaction with *Ureaplasma* spp. colonization. Preterm infants carrying this genotype who also had *Ureaplasma* spp. colonization faced a significantly increased risk of BPD (OR = 3.46, 95% CI: 1.58–7.61). Mechanistically, this polymorphism may modulate *TLR6*-mediated pathogen recognition and downstream NF-*κ*B signaling. Such alterations could influence host susceptibility and the intensity of the inflammatory response.

Intrinsic risk factors for *Ureaplasma* spp. infection in preterm infants primarily encompass low GA, low birth weight, abnormally elevated postpartum inflammatory markers (e.g., WBC, CRP), and host-related genetic polymorphisms. The underlying mechanism is likely attributable to the fact that infants with low GA and low birth weight possess immature immune functions and compromised skin/mucosal barriers. This physiological immaturity increases their susceptibility to *Ureaplasma* spp., rendering them more prone not only to colonization but also to progression to active infection (5). Furthermore, these infants are often subjected to prolonged hospitalization, frequent invasive procedures, and exposure to adverse intrauterine environments (e.g., PROM), which further exacerbates the risk of infection. Upon exposure to *Ureaplasma* spp., the host may mount a robust inflammatory response, characterized by increased secretion of pro-inflammatory cytokines such as IL-1β and IL-6. Consequently, dynamic monitoring of inflammatory markers in preterm infants holds critical clinical value for the risk assessment and management of *Ureaplasma* spp. infection (3).

## 3 Management of *Ureaplasma* spp. Infection in Preterm Infants

Whether antibiotic treatment is necessary for asymptomatic *Ureaplasma* spp. infection in neonates remains controversial. Some studies suggest that asymptomatic infection may not lead to serious complications and therefore does not require treatment (30). However, other studies advocate for early intervention to prevent potential complications, particularly in preterm infants whose immune systems are not yet fully developed. Given that this high-risk group is more susceptible to severe complications following infection, clinical practice tends to adopt an active prevention and treatment strategy (1). Early identification and treatment may be initiated when preterm infants present with risk factors such as low GA, low birth weight, elevated inflammatory markers, and poor response or lack of improvement with empirical anti-infective therapy, in the presence of maternal risk factors including vaginal delivery, PROM, or chorioamnionitis. In recent years, increasing research has focused on diagnostic techniques for the early identification of *Ureaplasma* spp. infection in preterm infants, as well as on the development of predictive models and management strategies. Therefore, it is necessary to systematically review the current progress and bottlenecks in predictive modeling and treatment strategies, with the aim of providing a theoretical basis for promoting precision therapy and safety management of *Ureaplasma* spp. infection in preterm infants.

### 3.1 Laboratory testing of *Ureaplasma* spp. Infection

Culture is the gold standard for detecting *Ureaplasma* spp. and can provide data on drug sensitivity. However, because *Ureaplasma* spp. lacks a cell wall and is sensitive to drying and high temperatures, false-negative results are likely if the specimen is collected in insufficient quantities, not sent promptly for testing, or improperly preserved (31). Molecular biology methods can achieve typing and grouping of the Ureaplasma genus (32); they require a small sample volume and offer the advantages of rapidity, high sensitivity, and high specificity (33). PCR technology is currently the main approach used. The sensitivity and PPV of the real-time fluorescence LAMP assay have been reported to be 100% (16/16 specimens; 95% CI: 79.4%–100%) and 100% (16/16; 95% CI: 79.4%–100%) compared to culture results (34). However, the PCR technique may produce false positives. Metagenomic next-generation sequencing, an emerging molecular biology technique based on high-throughput sequencing, has the advantages of being unbiased, having broad coverage, high sensitivity, and relatively rapid pathogen identification (35). Using metagenomic next-generation sequencing, Okumura et al. were the first to reveal that the presence of Ureaplasma in the gastric fluid of neonates with respiratory distress is associated with chorioamnionitis (36).

### 3.2 Predictive models for *Ureaplasma* spp. Infection

Predictive models incorporate prenatal risks and postnatal management indicators for preterm infants, providing important tools for precise prevention and treatment of *Ureaplasma* spp. infection. Viscardi et al. conducted a retrospective study on 415 preterm infants with GA < 33 weeks and developed an antenatal predictive model for *Ureaplasma* spp. infection in preterm infants using optimal subset regression (18). This model incorporated two perinatal indicators: GA and PROM duration > 72 h. The model expression was 8.872−(0.381) (GA) + (0.961) (PROM duration). The Area Under the Receiver Operating Characteristic Curve (AUC) was 0.73, suggesting that the model has a certain predictive ability for identifying preterm infants with lower respiratory tract *Ureaplasma* spp. infection, and the selected indicators are readily obtainable in clinical practice. Additionally, Viscardi et al. constructed two early postnatal predictive models to distinguish populations with low risk (< 10%) and high risk (> 40%) of lower airway Ureaplasma infection (18). Model 1 included GA, PROM > 72 h, and white blood cell count, with the expression: 7.699−(0.355) (GA) + (0.046) (WBC) + (0.763) (PROM duration), yielding an AUC of 0.77. Model 2 included GA, PROM > 72 h, and mode of delivery, with the expression: 9.503−(0.387) (GA) + (1.015) (PROM duration)−(0.732) (cesarean section), yielding an AUC of 0.75. Li et al. developed a predictive model based on data from 870 preterm infants with GA between 28 and 37 weeks (17). This model included three predictive factors: mode of delivery, GA, and CRP (> 0.5 mg/L). The model expression was 3.428−(0.163) (GA) + (1.136) (vaginal delivery) + (1.625) × (CRP), achieving an AUC of 0.800 (95% CI: 0.758–0.843), indicating good predictive performance. In contrast, Sun et al. constructed a model combining three factors—the mode of delivery, PROM, and leukocyte level—in 291 preterm infants with GA ≤ 32 weeks and birth weight ≤ 2000g, which yielded an AUC of 0.658 (19).

The indicators used in the above-mentioned models are highly clinically accessible, facilitating rapid risk stratification after birth and enabling primary healthcare institutions to achieve early identification and selective screening of high-risk populations. In terms of prevention and treatment strategies, accurately identifying high-risk infants reduces the indiscriminate use of empirical antibiotics, significantly improving the rationality of antibiotic application. Currently, although some predictive models have demonstrated good predictive performance, most still lack large-scale, multicenter external validation. Future efforts should focus on further optimizing these models by incorporating more comprehensive indicators, such as genetic polymorphisms, to enhance their clinical applicability and promote broader adoption.

### 3.3 Management of *Ureaplasma* spp. Infection in Preterm Infants

#### 3.3.1 Maternal management

Standardized treatment of maternal *Ureaplasma* spp. infection is a crucial strategy for interrupting vertical transmission and improving perinatal outcomes. However, currently, there is no unified clinical treatment protocol (37). Macrolide antibiotics (such as erythromycin, clarithromycin, and azithromycin) are safe options for treating *Ureaplasma* spp. infection during pregnancy. Among them, azithromycin possesses good tissue penetration and a long half-life. It can be actively taken up by macrophages, can target the infection site, and can exert an inhibitory effect on Ureaplasma spp. Therefore, it is considered the first-line treatment for *Ureaplasma* spp. infection during pregnancy. Considering the efficacy of azithromycin on *Ureaplasma* spp. at different doses and duration of administration, the meta-analysis showed that azithromycin had a comparable therapeutic effect compared to controls, whether given as a single dose of 1 g or 0.5 g once daily for 7 days (37). It is noteworthy that although prenatal azithromycin intervention can reduce the risk of moderate-to-severe bronchopulmonary dysplasia in preterm infants, it does not reduce respiratory tract *Ureaplasma* spp. colonization in preterm infants. This finding suggests that the protective effect of azithromycin may be more attributable to its anti-inflammatory properties rather than a direct bactericidal effect against *Ureaplasma* spp. (25). Preclinical studies indicate that a new macrolide-derived antibiotic—solithromycin has broad-spectrum intracellular bioactivity and greater transplacental transfer capacity (38). However, rigorous future clinical trials are needed to evaluate its safety and efficacy in pregnant women, as data are currently lacking.

Given the limitations of antibiotic treatment alone in completely clearing the pathogen and preventing adverse pregnancy outcomes, combination therapy strategies are gaining attention (39). Studies have shown that the combination of traditional Chinese medicine and antibiotics in treating female *Ureaplasma* spp. infection yields superior comprehensive efficacy and pathogen clearance rates compared to antibiotics alone, with good safety (40). Qiu et al. further pointed out that combining Chinese and Western medicine not only enhances efficacy and reduces toxicity but also helps delay the emergence of drug-resistant strains (41). Mechanistically, since *Ureaplasma* spp. lacks a cell wall, the active components of traditional Chinese medicine can penetrate and diffuse into the organism through semi-permeable cell membranes, thereby inhibiting the growth and increasing the death of the organisms. Moreover, these components inhibit *Ureaplasma* spp. growth by modulating host immunity and improving the internal environment. Furthermore, innate immune cells at the maternal-fetal interface (such as natural killer cells and macrophages) play a crucial role during pregnancy (42). Traditional Chinese medicine helps maintain the dynamic balance of maternal-fetal immunity, reduces excessive tissue cell apoptosis, and suppresses excessive inflammatory responses by attenuating cytotoxicity (43). Another study found that vaccination could become an effective medical intervention to prevent *Ureaplasma* spp. infection (44). A mouse model showed that intramuscular injection of recombinant Ureaplasma spp.-DnaJ induced a robust immune response, effectively preventing genital tract infection, reducing maternal inflammation, and mitigating pathological sequelae. Therefore, exploring comprehensive intervention strategies applicable to pregnant women, such as vaccination, integrated traditional Chinese and Western medicine, and immunomodulation, alongside conventional antibiotic therapy, holds promise for optimizing the management of prenatal maternal *Ureaplasma* spp. infection.

#### 3.3.2 Preterm infants management

To prevent adverse outcomes resulting from *Ureaplasma* spp. infection in preterm infants, implementing early screening and seizing the optimal timing for prevention and treatment is particularly urgent. However, currently, there is no unified consensus regarding the selection of anti-infective regimens, dosage, and administration for *Ureaplasma* spp. infection in preterm infants, necessitating high-quality research for clarification (12). Due to the immature organ development of preterm infants and the potential safety risks of related anti-infective drugs, clinical decision-making faces challenges in risk-benefit assessment, leading to considerable variation in clinical practice. Regarding drug selection, although quinolones, tetracyclines, or chloramphenicol are effective against *Ureaplasma* spp. infection, their use in preterm infants is limited due to potential adverse effects: chloramphenicol may cause gray baby syndrome and bone marrow aplasia; doxycycline may cause tooth discoloration and enamel hypoplasia; and quinolones carry a potential risk of tendon rupture. Currently, macrolides remain the mainstay for treating *Ureaplasma* spp. infection in preterm infants. Macrolides not only effectively clear *Ureaplasma* spp. infection but also exert anti-inflammatory and immunomodulatory effects through non-antibacterial mechanisms, such as inhibiting nuclear factor-*κ*B expression, reducing inflammatory cytokine release, and promoting cell apoptosis (37, 45).

There is insufficient research data supporting the efficacy of macrolides like erythromycin and clarithromycin in *Ureaplasma* spp. eradication (12), whereas the clinical advantages of azithromycin are more prominent. Viscardi et al. found that intravenous administration of azithromycin (20 mg/kg every 24 h) for 3 consecutive days in preterm infants resulted in a 100% respiratory tract *Ureaplasma* spp. clearance rate, higher than that of erythromycin (82%–86%) and clarithromycin (68.5%), with no observed adverse effects on long-term pulmonary and neurodevelopmental outcomes (30). Multiple studies indicate that azithromycin treatment helps reduce the incidence of bronchopulmonary dysplasia and mortality in *Ureaplasma* spp.-positive preterm infants and effectively shortens the duration of mechanical ventilation and oxygen therapy, but its clinical benefit is closely related to the dosing regimen (e.g., dose, duration, route of administration) (46–48). However, regarding the therapeutic evidence for azithromycin in preventing BPD in preterm infants, the double-blind, randomised, placebo-controlled trial by Lowe et al. yielded a discordant conclusion. The trial found that, regardless of the presence of *Ureaplasma* spp. colonization, a 10-day course of azithromycin (20 mg/kg once daily for the first 3 days followed by 10 mg/kg once daily for the remaining 7 days) failed to improve BPD-free survival in preterm infants (49). The above findings indicate that the clinical application of azithromycin still faces three major challenges: First, the optimal dosing regimen has not been established, current evidence suggests that different dose and duration combinations may lead to markedly different outcomes; therefore, large-scale, high-quality randomised controlled trials are urgently needed to determine safer and more effective regimens (46, 49); Second, its efficacy in treating central nervous system infections is controversial, although the drug can penetrate the blood-brain barrier and persist in tissues for a long time, its concentration in cerebrospinal fluid is relatively low, limiting its therapeutic potential for *Ureaplasma* spp. meningitis (1); finally, safety data have limitations. Potential risks such as hypertrophic pyloric stenosis, QT interval prolongation, and torsades de pointes have not been fully quantified in preterm infants (50).

## 4 Conclusions

*Ureaplasma* spp. infection is highly prevalent in preterm infants and often leads to adverse clinical outcomes. Its occurrence is influenced by multiple risk factors related to both the mother and the infant. The application of highly sensitive molecular biological testing techniques (such as PCR and metagenomic next-generation sequencing) can significantly improve the detection rate and typing accuracy of *Ureaplasma* spp., providing key evidence for early diagnosis and risk stratification. Building risk prediction models on this basis facilitates early identification of high-risk infants and timely intervention, which is of great significance for improving prognosis. Although macrolides remain the first-line clinical choice, challenges such as bacterial resistance and medication safety persist. Future efforts should focus on conducting multi-center, large-sample prospective studies, incorporating advanced testing techniques to develop more accurate predictive models, and exploring safer and more effective intervention strategies, including the integration of traditional Chinese and Western medicine, to comprehensively enhance the long-term quality of life of preterm infants.
